# Modulating Neural Circuits of Pain in Preclinical Models: Recent Insights for Future Therapeutics

**DOI:** 10.3390/cells13120997

**Published:** 2024-06-07

**Authors:** Juliette Viellard, Rabia Bouali-Benazzouz, Abdelhamid Benazzouz, Pascal Fossat

**Affiliations:** 1 Université de Bordeaux, UMR 5293, F-33076 Bordeaux, France; 2 CNRS, Institut des Maladies Neurodégénératives, UMR 5293, F-33000 Bordeaux, France

**Keywords:** pain matrix, brain stimulation, chronic pain

## Abstract

Chronic pain is a pathological state defined as daily pain sensation over three consecutive months. It affects up to 30% of the general population. Although significant research efforts have been made in the past 30 years, only a few and relatively low effective molecules have emerged to treat chronic pain, with a considerable translational failure rate. Most preclinical models have focused on sensory neurotransmission, with particular emphasis on the dorsal horn of the spinal cord as the first relay of nociceptive information. Beyond impaired nociceptive transmission, chronic pain is also accompanied by numerous comorbidities, such as anxiety–depressive disorders, anhedonia and motor and cognitive deficits gathered under the term “pain matrix”. The emergence of cutting-edge techniques assessing specific neuronal circuits allow in-depth studies of the connections between “pain matrix” circuits and behavioural outputs. Pain behaviours are assessed not only by reflex-induced responses but also by various or more complex behaviours in order to obtain the most complete picture of an animal’s pain state. This review summarises the latest findings on pain modulation by brain component of the pain matrix and proposes new opportunities to unravel the mechanisms of chronic pain.

## 1. Introduction

Chronic pain is one of the most common pathological syndromes, affecting up to one-third of the general population. Chronic pain can be either elicited by chronic inflammation, lesion of the central or peripheral nervous system or be idiopathic with no clear causes [1]. The current medication for chronic pain is based on the repositioning of two main drug families: anticonvulsants and antidepressants. These molecules are chronic treatments associated with NSAIDs and opioids for acute pain management [2,3]. Unfortunately, only a few patients achieve effective pain relief with these treatments, which also have significant side effects [3,4]. On the one hand, this led to an overprescription of opioids in the early 2000s associated with misuse responsible for the opioid crisis [5,6]. On the other hand, a large number of patients remain on the treatment without an alternative. Neurostimulation is also used to treat chronic pain and can be divided into two approaches: a non-invasive approach, which can be transcutaneous electrical nerve stimulation or transcranial magnetic stimulation, generating analgesia for chronic pain [7], and an invasive approach, which needs a surgical intervention for unilateral or bilateral implantation of a chronic electrode, for which the choice of a target and the precise implantation of the stimulating electrode are crucial. Spinal cord stimulation generates analgesia but also undesirable side effects such as paraesthesia [7]. It is restricted to patients suffering from intractable pain like failed back surgery syndrome or complex regional pain syndrome [7]. Brain stimulation can either be deep, when applied to deep brain structures such as the periaqueductal grey (PAG) and thalamus, or superficial, when applied to the motor cortex. The underlying mechanisms of analgesia are not fully established, but it is suggested that it involves endogenous opioids in the case of deep brain stimulation and ascending and descending pain control for the motor cortex stimulation [7].

Nociceptive transmission starting from the activation of specific receptors in peripheral nerve endings reaches the spinal cord and then projects to various types of supraspinal areas. It includes the brainstem, midbrain, basal ganglia and cortical areas.

Thanks to the number of studies using functional brain imaging, it has become clear that a combination of areas is responsible for pain processing [8,9]. These pain-activated brain regions are known as the pain matrix, which is now used to describe pain perception in the brain [10,11]. This intricate pain processing explains why pain sensation is such a complex phenomenon, difficult to manage and control. Moreover, all brain regions participating in pain sensation are not exclusively dedicated to pain processing but are also involved in other sensory, motor and cognitive functions [12]. Pain sensation can be divided into three different components involving specific but intermingled cortical and subcortical areas that compose the “pain matrix”. Firstly, the sensory-discriminative component of pain allows a precise localisation and detection of pain and its intensity. Secondly, the emotional component will usually associate a negative emotion with the pain sensation, worsening the latter. Finally, the cognitive component will complete the pain sensation by associating memory from past experiences that could either worsen or improve the experience. In addition, subcortical areas relay nociceptive information concurrently, generating cortical and subcortical loops that modulate pain signals and associated emotions. Stimulating one or several elements of the “pain matrix” can help to treat chronic pain symptoms by acting on one or several of the three components of pain sensation [13]. We propose to review the state-of-the-art techniques in preclinical studies manipulating the brain regions involved in the “pain matrix”. We will first describe the regions of interest based on human imaging, and then, we will focus on the consequences of the manipulation of the main regions of the “pain matrix” in preclinical models, including the ones involved in sensory, cognitive and emotional pain. This will allow us to identify potential therapeutic avenues for future treatments of chronic pain. The recent advances in the field have given new crucial insights to improve the efficacy of brain stimulations in the future.

## 2. Pain Imaging in Humans

**Figure 1:**
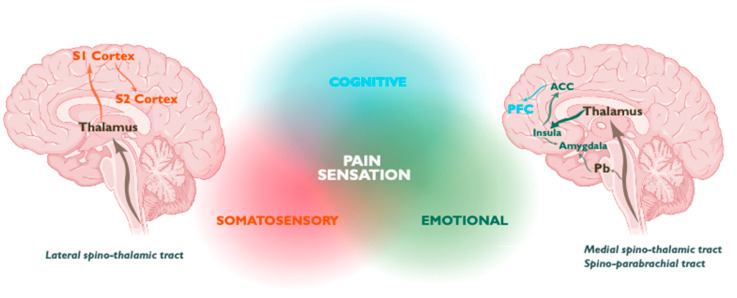
Scheme of the main ascending pathways for pain perception. S1: primary somatosensory cortex; S2: secondary somatosensory cortex; ACC: anterior cingulate cortex; PFC: prefrontal cortex; Pb: parabrachial nucleus. The spino-thalamic tract of the lateral pathway controls the somatosensory component of pain (in red). The spino-thalamic tract of the medial pathway associated with the spino-parabrachial tract controls the emotional component of pain (in green).

In the last decades, human imaging techniques have improved so much that numerous advances have shed light on the brain structures involved in the sensation of pain. The main regions activated following a noxious stimulus are the primary somatosensory cortex (S1), the secondary somatosensory cortex (S2), the anterior cingulate cortex (ACC), the insula, the prefrontal cortex (PFC), the thalamus and the cerebellum [14]. These structures are involved in different brain functions comprising emotions (ACC and insula), cognition (PFC) and somato-sensation (S1). They are not activated at the same time, and there is a progressive activation of cortical areas before and after conscious pain perception. More precisely, the posterior insula, operculum, midcingulate cortex and amygdala are activated before conscious pain, and the anterior insula and prefrontal cortex are activated during pain consciousness, suggesting that noxious stimuli first reach the emotional areas before the cognitive ones [15,16,17]. Moreover, different pain pathways seem to be responsible for pain sensation, a spinothalamic tract comprising a lateral pathway projecting to the brain through the lateral thalamus, including S1 and S2, a medial pathway reaching ACC and the insula, then PFC through the medial thalamus (MD) [12] and a spino-parabrachial tract reaching the amygdala (Figure 1) [16]. In pathological pain, a reorganisation of the “pain matrix” appears but differs based on the type of pathological state. For instance, in osteoarthritis patients, pain is associated with the activation of the cingulate cortex, amygdala and putamen, all brain regions that are linked to responses to aversion, reward and fear [18]. In patients with neuropathic pain and expressing dynamic allodynia, an alteration of the lateral pathway (increased activity in S1 or S2 or PFC) is more prominent than a modification of the medial pathway (decreased activity or no change in the ACC and insula), suggesting an alteration of the somatosensory rather than the emotional component of pain [19,20,21,22,23]. However, patients with peripheral mononeuropathy express an overactivation of the entire “pain matrix” [24,25]. In conclusion, brain regions involved in the “pain matrix” are separated into different clusters receiving nociceptive inputs from either the lateral or medial thalamus and parabrachial nuclei and processing the sensory-discriminative and affective-emotional component of pain, respectively (Figure 1) [12]. A third cluster of brain structures will manage the cognitive component (Figure 1) [26,27]. These pathways are associated with the midbrain and brainstem regions that participate in the descending control of pain, such as the periaqueductal grey (PAG), the rostral ventromedial medulla (RVM), the locus coeruleus (LC), the parabrachial nucleus and the cerebellum [15]. Pathological pain modifies pain processing of all or part of this “pain matrix”, and discrepancies depend on individuals, the types of pathologies and the types of stimuli [15]. Future challenges will be to determine a precise cartography of the brain regions activated in a specific pathological context, associated with a specific category of patients. However, in recent years, the different components of the “pain matrix” have been studied in preclinical models using new technological approaches with unprecedented spatial and temporal resolution. In the next section, we will describe the recent advances in preclinical models, unravelling the contribution of each “pain matrix” component in pain transmission and control.

## 3. Contributions of Optogenetics and Chemogenetics

Recently, new methodologies have allowed considerable improvement in the knowledge of neural networks in general and pain circuits in particular. These methodologies were developed in the mid-2000s and are now widely and routinely used in preclinical models of pain. The first method is called optogenetics and allows a precise spatial and temporal manipulation of specific neuronal circuits [28]. It is usually based on the use of transgenic animals and virus-based opsin expression. The goal is to target a specific population of neurons aimed by an optic fibre connected to a LED or a laser that activates an opsin, either channel rhodopsin or archaerhodopsin (ChR2 or ArchT (or Arch3.0), or halorhodopsin that, in turn, activates or inhibits the targeted neuronal population [29]. For instance, optogenetic neuronal stimulations can efficiently increase brain activity and response to sensory inputs [30]. The second technique is called chemogenetics and is based on the use of Designer Receptor Exclusively Activated by Designer Drugs (DREADD) expressed in a specific neuronal population [31]. DREADD are expressed using also a virus-based approach allowing the expression of DREADD, a G-protein-coupled receptor (GPCR) that can be either excitatory (hM3Dq) or inhibitory (hM4Di). clozapine-N-oxide (CNO) or a selective agonist deschlorozapine (DCZ), synthetic molecules designed to specifically activate DREADD, is injected and activates the targeted neuronal population. Although the timing is less controlled than in optogenetics, DREADD agonists can be orally, intraperitoneally, intravenously or locally applied and act over a few hours. The use of DREADD requires less equipment, is less invasive and the long-lasting action of the agonists is suitable for behavioural approaches [32,33].

## 4. Manipulating the Brain “Pain Matrix”

### 4.1. Sensory-Discriminative Component

#### 4.1.1. Somatosensory Cortex

The somatosensory cortex S1 receives information from all peripheral areas of the body, including nociceptive inputs. This area is involved in the localisation and intensity of the message received. It has been demonstrated in humans and rodents that a specific gamma oscillation rhythm in the S1 cortex is correlated with pain perception and intensity [34]. A recent study used an optogenetics strategy to modulate the inhibitory parvalbumin interneurons (PVs) in the cortex S1, a modulation known to promote gamma oscillations and synchronisation of pyramidal neurons [35]. Using PV-cre mice, the authors expressed the excitatory opsin ChR2 to activate PV neurons in the gamma band frequency at 40 Hz or 80 Hz. This optical stimulation promoted hypersensitivity to a mechanical stimulation and placed aversion, suggesting that producing gamma oscillations in the S1 somatosensory cortex promotes pain sensitisation and contributes to the emotional component of pain. This effect is mediated by descending pain pathways controlling spinal nociceptive transmission, with a serotonergic relay in the RVM [36]. The same group showed that the inhibition of excitatory neurons of the cortex S1 suppressed pain hypersensitivity following capsaicin injection. The activation of pyramidal neurons of layers 5 and 6 in the S1 cortex have antagonist actions on the mechanical withdrawal threshold. Furthermore, the activation of layer 6 is pronociceptive through an increased activation of the thalamus and an inhibition of the layer 5 pyramidal neurons, whereas layer 5 activation is analgesic [37].

#### 4.1.2. Mid-Cingulate Cortex

The mid-cingulate cortex (MCC) is one of the first brain areas activated following noxious stimulation, but its precise role in pain processing is still poorly known. A recent study has shown that optogenetic inhibition of excitatory neurons of the MCC suppressed pain hypersensitivity induced by capsaicin injection in the hind paw. The authors showed that the role of MCC is, at the onset of pain sensitisation, silencing the MCC during the first 15 min of capsaicin action-blocked pain sensitisation. Moreover, the MCC is also important for the maintenance of sensitisation by silencing MCC 30 min after capsaicin-blocked sensitisation 1 h after capsaicin injection [38].

### 4.2. Cognitive and Emotional Components of Pain

#### 4.2.1. Anterior Cingulate Cortex (ACC)

The ACC is a brain region involved in pain and mood behaviours, known to be activated during conscious pain sensation [14]. Interestingly, ACC lesions in a preclinical model of neuropathic pain suppressed anxio-depressive behaviours [39], while an optogenetic activation of the area induced place aversion [38]. Altogether, these results suggest that the ACC is involved in the emotional component of pain without impacting in its sensory component. However, a recent study has shown that the ACC stimulation produced an increase in the excitability of wide dynamic range (WDR) neurons of the dorsal horn of the spinal cord (DHSC) involving direct ACC projections to the spinal cord [40]. The optogenetic activation of ACC projecting neurons to the spinal cord elicited, in this case, pain hypersensitivity. Interestingly, after a nerve injury, the ACC stimulation did not induce pain hypersensitivity or neuronal hyperexcitability, while an optogenetic inhibition reduced pain hypersensitivity in the same model but not in naïve animals. These results suggest that ACC plasticity induced in neuropathic pain is partly responsible for the increase in nociceptive transmission at the level of the spinal cord. Furthermore, a recent study has shown that the activation of the circuit ACC–posterior insula (PI) also increases nociceptive transmission, dorsal neuron excitability in the DHSC and mechanical pain sensitivity [41]. In summary, the ACC is involved in the emotional component of pain but also controls its sensory component through direct or indirect actions on nociceptive transmission at the level of the DHSC.

#### 4.2.2. Prefrontal Cortex (PFC)

This structure participates in social cognition, conceptualisation and working memory and is also activated in humans following painful stimulation during a conscious pain period after noxious stimuli [12,38]. The optogenetic modulation of excitatory outputs from the PFC have been explored. Particularly, projections from the prelimbic area of the PFC to the PAG participate in descending pain inhibition but are restricted to pathological pain. Indeed, the optogenetic activation or inhibition of PFC–PAG projections has no effect on the mechanical or thermal threshold in naïve animals. In contrast, the optogenetic activation of this circuit after peripheral nerve injury reduced pain sensitivity. Interestingly, after peripheral nerve injury, this network is tonically activated, since its optogenetic inhibition worsened the pain hypersensitivity [42]. The PFC–PAG circuit is controlled locally in the PFC by parvalbumine (PV) GABA inhibitory neurons. PV optogenetic activation inhibits pyramidal neurons and increases pain sensitivity, while silencing those neurons generates analgesia [43]. PFC–PAG projections mediate analgesia after a peripheral nerve lesion through serotonin and noradrenaline descending the inhibition [42]. In contrast, a recent study showed that a PFC neuronal ensemble can generate pain hypersensitivity. Unexpectedly, this neuronal ensemble is composed by mainly glutamatergic neurons and activated by painful stimuli, producing pain hypersensitivity through projections to the basolateral amygdala (BLA), nucleus accumbens and PAG [44]. Finally, all these studies show that PFC manipulation affects pain sensitivity and anxio-depressive behaviour, suggesting that PFC acts through both sensory and emotional pain components [43,44]. Altogether, these results suggest that brain stimulation of the cortical areas can either increase or reduce pain sensation, with the S1, S2 or ACC stimulation pronociceptive, while PFC stimulation can be either pronociceptive or antinociceptive, depending on the context and the downstream projections (Table 1). Because all these structures are activated by painful stimulation, an interesting approach to relieve intractable neuropathic pain with conventional medication would be to develop a closed-loop approach. A closed loop is a system or device that automatically regulates a system and that works independently of any human intervention. In pain context, the device would detect brain regions of overactivity associated with pain and, in turn, would activate the region that elicits pain relief. In the “pain matrix”, it would detect electrophysiological pain biomarkers in the ACC, S1 or S2, and, in turn, stimulate the PFC to limit pain sensation using this closed-loop brain machine interface. Recent publications have illustrated this possibility by developing a complex brain machine interface that detects electrophysiological pain biomarkers at the level of the S1 and ACC and, in turn, triggers prefrontal cortex stimulation to relieve pain with a closed-loop system [45,46]. The authors demonstrated that the brain machine interface improves pain symptoms as efficiently as human-directed manipulations [45].

#### 4.2.3. Amygdala

The amygdala is known for its role in emotions, and for almost 20 years, many publications have demonstrated its role in the emotional components of pain (for review, see [64] (Figure 1)). Simply put, nociceptive inputs reach the central nucleus of the amygdala, the lateral amygdala receives nociceptive inputs from the cortical areas, including the insula, and the BLA receives inputs from the PFC. Highly processed nociceptive information is then sent back to the central nucleus of the amygdala (CeA) that, in turn, projects to the descending pain control nuclei of the brainstem [65]. The BLA is mainly composed of glutamatergic neurons, and an optogenetic stimulation of the excitatory projections from the BLA to the PFC promoted pain hypersensitivity, while its inhibition decreased pain sensitivity in neuropathic animals [42]. In another study, the authors used the TRAP technique (Targeted Recombination in Active Populations) to isolate neuronal populations in the BLA that are activated specifically after noxious stimulations. Inhibiting that specific population reduced the aversive (emotional) component of pain, while the sensory component remained unaffected [52]. Moreover, optogenetic activation of BLA projections to the ACC reduced the aversive pain component, while its inhibition promoted aversion in a neuropathic pain model [47]. Altogether, these studies suggest that BLA modulation of pain depends on its projection to the cortex.

The CeA is also involved in pain detection. Indeed, the lateral and capsular division of the CeA receive exclusively nociceptive inputs, notably from the parabrachial nucleus. This area is named the “nociceptive amygdala” [66]. In contrast to the BLA, the role of the CeA in pain control is more uncertain. Recent studies have shown that inhibitory neurons from the CeA project to the vlPAG and inhibit GABA neurons. These GABA neurons tonically inhibit glutamate neurons that promote descending pain inhibition [50]. In another study, the activation of CeA neurons through projecting BLA neurons generated hyperalgesia [51].

#### 4.2.4. Insular Cortex

The posterior insula (PI) is also a crucial cortical area involved in the sensory component of pain. It is activated following a painful stimulation before conscious pain. The posterior insula receives projections from other cortical and subcortical areas and projects to the brainstem nuclei belonging to the pain descending controls. In recent studies, activation of the posterior insula induced pain hypersensitivity by activating facilitatory descending pain control pathways [38,41]. It has been reported in a model of chronic neuropathic pain that PI was involved in the sensory component of pain without altering the emotional component [39].

### 4.3. Subcortical Areas Pathways

#### 4.3.1. Thalamus

The thalamus is a relay of nociceptive inputs from the periphery to the cortex. Increasing evidence strongly supports a more complex role of the thalamus in pain perception [67]. Mostly, a decreased activity of the thalamus is associated with an increase in pain perception. This is particularly the case for intractable pain associated with the “thalamic syndrome”, a pathology elicited by central post-stroke pain [68]. Ascending nociceptive pathways project to the three main regions of the thalamus, as 35% of the lateral thalamus, including ventral posterior lateral (VPL) and ventral posterior inferior (VPI), projects to the S1 and S2 somatosensory cortexes, 40% to the posterior thalamus and 25% to the MD [67,69]. Optogenetic modulations of the MD have recently enlightened that activating MD projections to the ACC increased the emotional pain component in neuropathic animal models. Conversely, inhibiting that same pathway decreased the emotional component of pain, whatever the conditions [47].

#### 4.3.2. Descending Pain Pathways

The midbrain and brainstem nuclei are the main relays for the descending pain controls, particularly the PAG and the RVM of the reticular formation. It has been known since the early 1970s that electrical stimulation of the PAG, particularly the ventrolateral part (vlPAG), mediates analgesia [70,71]. The chemogenetic activation of the vlPAG generates an alteration of sensory pain with a moderate analgesic action, while its inhibition is proalgesic [53]. The analgesic effect of vlPAG activation is mediated by glutamatergic excitatory output neurons, while inhibitory interneurons facilitated sensory pain [50,53,55]. However, PAG also controls motor output to the spinal cord, notably associated with fear, and it is difficult to discriminate which neuronal population targets pain or the motor output. This is particularly the case for excitatory glutamatergic neurons of the PAG [50,56]. In contrast, dopamine neurons of the vlPAG/dorsal Raphe have a pure analgesic action and serve as potential targets of DBS therapy [56].

In a recent study, the authors developed biocompatible stimulating microelectrodes that precisely activate analgesic areas of the PAG without altering the motor areas [72]. PAG projects to the RVM, the second relay of the descending pain pathway. The RVM nucleus exerts both an excitatory and inhibitory barrage on nociceptive transmission at the level of the dorsal horn of the spinal cord. Facilitation or inhibition of this pathway depends not only on the neurochemical nature of the stimulated neurons but also on their electrophysiological signatures responding to nociceptive stimuli. For instance, chemogenetic or optogenetic activation of GABAergic neurons projecting to the spinal cord have a facilitating action on nociceptive transmission. This descending facilitation is due to the inhibition of spinal inhibitory enkephalin expressing neurons [57]. Optogenetic activation of serotonergic neurons of the RVM have bidirectional effects either facilitating or inhibiting nociceptive transmission, depending on the intensity of the stimulation and the pathophysiological context. High-frequency stimulation or stimulation in neuropathic models facilitate nociceptive transmission, while low-frequency stimulation in naïve animals inhibits nociceptive transmission [58,60]. Classically, noradrenergic inputs (NAs) from the LC to the spinal cord are considered antinociceptive, and their action is known to be mediated by α2-receptors [73]. However, recent studies, using optogenetics, revealed that the noradrenergic system is more likely to exert a bidirectional effect on pain transmission. Indeed, the optogenetic activation of noradrenaline projecting neurons induced both anti- and pronociceptive actions, depending on their localisation in the dorsal or ventral LC [62]. NA neurons projecting to the spinal cord inhibited DHSC neurons through the α1-receptor but also suppressed the diffuse noxious inhibitory control (DNIC) via descending inhibition involving α2-receptors [61]. Finally, the α1-receptor-mediated circuit in the superficial lamina of the DHSC is involved in pronociception through the activation of astrocytes [63]. Targeting the descending pain pathways using DBS to control pain needs a precise spatiotemporal pattern to efficiently relieve pain without triggering unwanted side effects.

## 5. New Pain Behaviour Assessments

All these reported studies of the literature measured the different components of pain, mainly by focusing on evoked pain measurements in ethologically unnatural conditions [74]. Usually, to assess the sensory components of pain, i.e., nociception, the reflex threshold is used to evaluate the force (mechanical) or the temperature generating nociceptive responses. The spontaneous pain is generally assessed through licking and flinching behaviours after nociceptive challenges with irritant molecules (capsaicin and formalin). The emotional component of pain is evaluated through aversive behaviours, place preference or avoidance/escape tests. However, these measures also include a reward/punishment component that is not fully related to pain [74]. Finally, recent publications have developed new, more ecological behavioural paradigms that could provide more appropriate information on the actual pain state of animal models. These paradigms will help make translational approaches more efficient in the future. This is the case of a recent study in which researchers filmed mice in their home cages and measured different behavioural features to correlate the occurrence of each behavioural feature and the level of pain [75]. Among all of the behavioural features assessed, the cage lid hanging behaviour was strongly correlated with mechanical allodynia in pain models and strongly reduced. Although this “natural” behaviour is of interest, several limitations have to be taken into account before deploying it more widely in pain studies. Indeed, cage lid hanging depends on the mouse strain, age of mice and time of day (mostly present at night), with a difference between females and males [75]. In other recent studies, the authors developed a maze where mice are encouraged to cross a corridor with nociceptive pins or temperature (hot and cold) [76]. In this case, the mouse has the choice between staying in the aversive chamber and slowly crossing the corridor, avoiding pinpricks, or quickly leaving the aversive chamber, ultimately walking on the nociceptive pins. Furthermore, in another recent study, they used an innovative concept to exploit deep learning strategies as a tool to identify facial characteristics of the mouse that can be associated with a pain-specific grimace [77]. In the future, the development of AI will allow us to assess more ecological behaviours and identify specific behavioural traits associated with pain by analysing a large quantity of data. This will lead to better understanding pain expression in animal models that, unlike humans, cannot verbally express their pain.

## 6. Conclusions

Recent advances in brain imaging allowing functional study of the brain in painful contexts highlighted the role of two different pathways, lateral and medial, which, respectively, process the sensory-discriminative and the affective-emotional components of pain. Altogether, specific neuroanatomical target of the cortical and subcortical areas can be an alternative to classical medication to erase intractable pain. Of particular interest for stimulation would be the PFC, the thalamus or the PAG or emotional-related areas such as the CeA. In contrast, the ACC, S1 and S2 and insula seem mainly to exert a proalgesic action. In the future, the use of proalgesic structures as biomarkers of pain activation could be useful to activate analgesic areas and generate pain relief “on demand”. Such closed-loop systems are of particular interest for future therapeutics, including both invasive and non-invasive strategies.

## Figures and Tables

**Table 1:** Description of the stimulation parameters of the different brain areas involved in pain perception and control (S1: somatosensory cortex; PI: posterior insula; PFC: prefrontal cortex; ACC: anterior cingulate cortex; CeA: central nucleus of the amygdala; BLA: basolateral amygdala; PAG: periaqueductal grey; RVM: rostral ventromedial medulla; LC: locus coeruleus; MCC: mid-cingulate cortex; PV: parvalbumine; GABA: gamma aminobutyric acid; Arch: archaerhodopsine; ChR2: channel rhodopsine; CNO: clozapine-N-oxide).

Pain Component	Brain Area	Type of Stimulation	Method	Protocol	Pain Outcome	References
Sensory	S1	Inhibition of excitatory neurons	Optogenetic ArchT	Continuous light	Mechanical hypersensitivity only with MCC stimulation	[38]
		Inhibition of excitatory neurons	Optogenetic ArchT	Continuous (minutes) or intermittent light	Prevent Capsaicin hypersensitivity	[38]
		Activation of L6	Optogenetic ChR2	Continuous 5 or 20 Hz	Mechanical hypersensitivity Aversive response	[37]
		Activation of L5	Optogenetic ChR2	Continuous 5 s or 20 Hz	Reduce Mechanical sensitivity No Aversive response	[37]
		Inhibition of L5	Optogenetic stGtACR2	Continuous 5 s or 20 Hz	Mechanical hypersensitivity	[37]
		activation of PV neurons	Optogenetic ChR2	40 or 80 Hz	Mechanical hypersensitivity Aversive response	[36]
	Thalamus	Activation MD-ACC	Optogenetic ChR2	20 Hz/5 ms	Light Aversion	[47]
		Inhibition MD-ACC	Optogenetic Arch	Continuous	Light Preference	[47]
Cognitive	Posterior Insula	Lesion of PI	Cell death	NA	Mechanical hypersensitivity	[39]
		Activation of MCC-PI	Optogenetic ChR2	30 Hz	Mechanical hypersensitivity	[38]
		Activation ACC-PI	Optogenetic ChR2	2 Hz/5 ms	Mechanical hypersensitivity	[41]
	PFC	Activation of PFC-vlPAG	Optogenetic ChR2	Continuous (minutes)	Mechanical and thermal analgesia in neuropathic pain model	[42]
		Inhibition of PFC-vlPAG	Optogenetic NpHR	Continuous (minutes)	Mechanical hypersensitivity in neuropathic pain	[42]
		Activation of PFC PV Inhibition of PFC PV	Optogenetic ChR2 Optogenetic Arch3.0	40 Hz/10 ms Continuous	Hyperalgesia in neuropathic animals Analgesia in neuropathic animals	[43]
		Activation of excitatory ensembles of PFC	Chemogenetic, HDM3q	CNO	Mechanical hypersensitivity	[44]
	Orbitofrontal Cortex	Activation of glutamate neurons	Chemogenetic HDM3q	CNO	Mechanical analgesia in neuropathic pain	[48]
		Activation of excitatory neurons	Optogenetic ChR2	10 Hz	Mechanical analgesia in neuropathic pain	[48]
Emotional	ACC	Activation	Electrical stimulation		Mechanical hypersensitivity	[40]
		Activation of inhibitory neurons of ACC	Optogenetic ChR2	10 Hz/20 ms	Decrease spontaneous pain behaviour	[49]
		Activation of ACC-SC	Optogenetic ChR2	20 Hz/40 ms	Mechanical hypersensitivity	[40]
		Inhibition of ACC-SC	Optogenetic Arch	Continuous	Mechanical analgesia in neuropathic pain	[40]
		Activation of ACC-PI	Optogenetic ChR2	2 Hz/5 ms	Mechanical and thermal hypersensitivity	[41]
		Inhibition of ACC-PI	Optogenetic ArchT	Continuous	Mechanical and thermal analgesia	[41]
	CeA	Activation of CeA-PAG	Not tested	Not tested	Thermal analgesia	[50]
		Activation of BLA to CeA	Optogenetic ChR2	20 Hz	Increase activity of spinal neuron	[51]
		Inhibition of CeA output neurons	Optogenetic NpHR3.0	Continuous	Decrease activity of spinal neurons	[51]
	BLA	Inhibition of BLA	Chemogenetic	CNO	Place preference	[52]
		Activation of BLA to PFC	Optogenetic ChR2	10 Hz	Mechanical and thermal hyperalgesia in neuropathic pain	[42]
		Inhibition of BLA to PFC	Optogenetic NpHR3.0	Continuous	Mechanical and thermal analgesia	[42]
		Activation of BLA to ACC	Optogenetic ChR2	20 Hz/5 ms	Place preference in neuropathic pain	[47]
Descending Pathways	PAG	vlPAG activation	Chemogenetic	CNO	Thermal analgesia	[53]
		vlPAG inhibition	Chemogenetic	CNO	Mechanical and thermal hyperalgesia	[50]
		vlPAG Glutamate neurons activation	Optogenetic ChR2 Chemogenetic	NA CNO	Mechanical and thermal analgesia	[50,53]
		vlPAG GABA neurons activation	Chemogenetic	CNO	Mechanical and thermal hyperalgesia	[53]
		vlPAG SST neurons	Chemogenetic	CNO	Mechanical and thermal hyperalgesia	[54]
		vlPAG SST neurons activation	Optogenetic ChR2	2 Hz/5 ms	Increased activity of spinal neurons	[55]
		vlPAG dopamine neurons activation	Chemogenetic	CNO	Mechanical and thermal analgesia	[56]
	RVM	Activation of GABA neurons	Optogenetic ChR2	15 Hz	Mechanical hypersensitivity	[57]
		Activation of 5HT neurons	Optogenetic ChR2 chemogenetic	5 Hz/5 ms CNO	Mechanical and thermal analgesia	[58,59]
		Activation of 5HT neurons	Optogenetic ChR2	20 Hz/15 ms 5 Hz/5 ms	Mechanical and thermal hyperalgesia in neuropathic pain	[58,60]
	LC	Activation of LC and LC to SC	Optogenetic ChR2	5 Hz/20 ms	Decreased spinal neuro response	[61]
		Dorsal LC	Optogenetic ChR2	Continuous	Thermal analgesia/hyperalgesia	[62]
		LC-SC	NA	NA	Hyperalgesia	[63]
